# Angiotensin (1-7) Decreases Myostatin-Induced NF-κB Signaling and Skeletal Muscle Atrophy

**DOI:** 10.3390/ijms21031167

**Published:** 2020-02-10

**Authors:** Javier Aravena, Johanna Abrigo, Francisco Gonzalez, Francisco Aguirre, Andrea Gonzalez, Felipe Simon, Claudio Cabello-Verrugio

**Affiliations:** 1 Laboratory of Muscle Pathology, Fragility and Aging, Department of Biological Sciences, Faculty of Life Sciences, Universidad Andres Bello, Santiago 8370146, Chile; 2 Millennium Institute on Immunology and Immunotherapy, Santiago 8370146, Chile; 3 Center for the Development of Nanoscience and Nanotechnology (CEDENNA), Universidad de Santiago de Chile, Santiago 8350709, Chile; 4 Millennium Nucleus of Ion Channels-Associated Diseases (MiNICAD), Universidad de Chile, Santiago 8370146, Chile; 5 Laboratory of Integrative Physiopathology, Department of Biological Sciences, Faculty of Life Sciences, Universidad Andres Bello, Santiago 8370146, Chile

**Keywords:** RAS, Angiotensin-(1-7), muscle atrophy, NF-κB signaling, Akt/PKB

## Abstract

Myostatin is a myokine that regulates muscle function and mass, producing muscle atrophy. Myostatin induces the degradation of myofibrillar proteins, such as myosin heavy chain or troponin. The main pathway that mediates protein degradation during muscle atrophy is the ubiquitin proteasome system, by increasing the expression of atrogin-1 and MuRF-1. In addition, myostatin activates the NF-κB signaling pathway. Renin–angiotensin system (RAS) also regulates muscle mass. Angiotensin (1-7) (Ang-(1-7)) has anti-atrophic properties in skeletal muscle. In this paper, we evaluated the effect of Ang-(1-7) on muscle atrophy and signaling induced by myostatin. The results show that Ang-(1-7) prevented the decrease of the myotube diameter and myofibrillar protein levels induced by myostatin. Ang-(1-7) also abolished the increase of myostatin-induced reactive oxygen species production, atrogin-1, MuRF-1, and TNF-α gene expressions and NF-κB signaling activation. Ang-(1-7) inhibited the activity mediated by myostatin through Mas receptor, as is demonstrated by the loss of all Ang-(1-7)-induced effects when the Mas receptor antagonist A779 was used. Our results show that the effects of Ang-(1-7) on the myostatin-dependent muscle atrophy and signaling are blocked by MK-2206, an inhibitor of Akt/PKB. Together, these data indicate that Ang-(1-7) inhibited muscle atrophy and signaling induced by myostatin through a mechanism dependent on Mas receptor and Akt/PKB.

## 1. Introduction

Skeletal muscle atrophy is the loss of muscle mass and function [1,2]. Several causes that induce skeletal muscle atrophy include immobilization, sepsis, aging, or chronic diseases [2,3]. Some features of skeletal muscle atrophy include the decrease of muscle strength and fiber diameter, and the diminution of myofibrillar proteins, such as myosin heavy chain (MHC) and troponin [4,5]. The latter is produced by an unbalance between catabolic and anabolic mechanisms. Thus, in skeletal muscle atrophy, a decreased synthesis and/or increased degradation is observed [5]. The main protein catabolic pathway is the ubiquitin proteasome system (UPS), which is increased under atrophic conditions, as evidenced by an increase in the gene expression of two muscle-specific E3 ubiquitin ligases, atrogin-1/MAF-bx and MuRF-1/TRIM63 [6]. These E3 ligases act for modifying MHC and troponin with ubiquitin [7]. In addition, the presence of oxidative stress is another mechanism involved in muscle atrophy. In general, oxidative status is caused by an increase of reactive oxygen species (ROS) production and/or a decrease of the antioxidant mechanism in cells, which causes cellular damage [8].

Among the soluble factors that induce muscle atrophy is myostatin/GDF8 [9,10]. Evidence shows that a decrease in the gene expression or activity of myostatin induces an increase in muscle mass and hypertrophy [11,12]. Similarly, the increase of myostatin levels induces muscle atrophy [10]. Myostatin is a growth factor that belongs to the transforming growth factor (TGF) family of proteins. The binding of myostatin to its transducer receptors mainly activates the Smad-dependent signaling pathway, which regulates the expression of the target gene [10]. In addition, myostatin activates NF-κB signaling, which is associated with the TNF-α (Tumor Necrosis Factor type alpha) gene expression [13]. Muscle atrophy induced by myostatin is evidenced by the increase of the MuRF-1 and atrogin-1 gene expressions and of the protein breakdown [14,15].

Another relevant regulator of skeletal muscle mass is the renin–angiotensin system (RAS) [16]. RAS can be functionally separated into classical and non-classical axes. The deleterious effects of the classical axis on skeletal muscle are associated to its main peptide angiotensin II (Ang-II) via the AT-1 receptor [17,18,19,20]. On the contrary, Angiotensin-(1-7) (Ang-(1-7)), the main peptide of the non-classical axis, produces a beneficial effect on skeletal muscle, such as a reduction of fibrosis and atrophy [5,21]. The effects of Ang-(1-7) on several tissues, including skeletal muscle, are produced through the G protein-coupled receptor Mas [22,23,24]. One of the signaling pathways activated by Ang-(1-7) via the Mas receptor is the IGF-1 (Insulin-like Growth Factor 1)/IGF-1R/Akt/PKB (Protein Kinase B) [25]. To date, the role of Ang-(1-7) on skeletal muscle atrophy induced by myostatin and the mechanisms that can participate have not been evaluated.

## 2. Results

### 2.1. Ang-(1-7) Prevents the Decrease of the Myotube Diameter and MHC Levels Induced by Myostatin in C_2_C_12_ Cells

**Figure 1:**
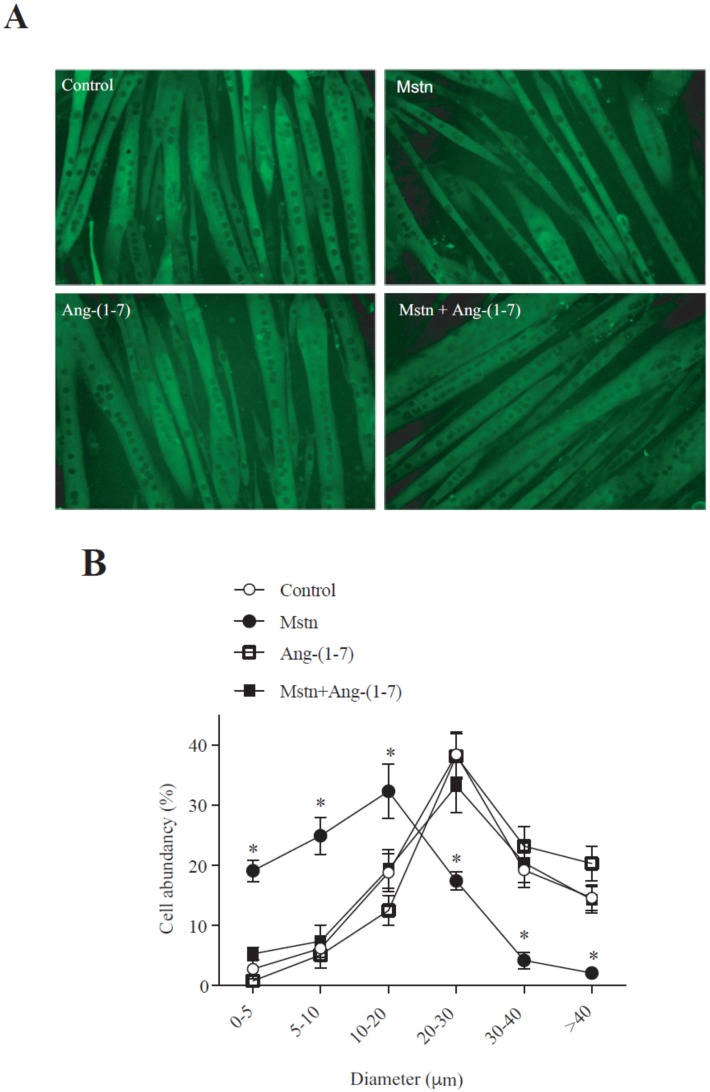
Angiotensin (1-7) (Ang-(1-7)) prevented the myostatin-induced decrease of the myotube diameter. (**A**) C_2_C_12_ myoblasts differentiated for five days (myotubes) were pre-incubated in the absence or presence of Ang-(1-7) (10 nM) for 60 min, and then incubated with myostatin (Mstn, 1 µg/mL) for 72 h. Myosin heavy chain (MHC; green) was detected through indirect immunofluorescence (IFI). Hoechst was used to stain the nuclei (blue). The bar scale represents 100 μm. (**B**) The graphics show the distribution of the myotube diameters. The values are expressed as a percentage of the total myotubes and correspond to the mean ± standard deviation (SD) from three independent experiments (*n* = 3; * *p* < 0.05 vs. control).

We analyzed the effect of Ang-(1-7) on the decrease of the C_2_C_12_ myotube diameter induced by myostatin. Figure 1A shows the myotube diameter delineated by the detection of MHC via indirect immunofluorescence. The quantification of these data indicates that myostatin induced a displacement to the left in the size’s distribution of the myotubes (Figure 1B). The same figure shows that Ang-(1-7) avoids myostatin-induced displacement towards decreased sizes.

**Figure 2:**
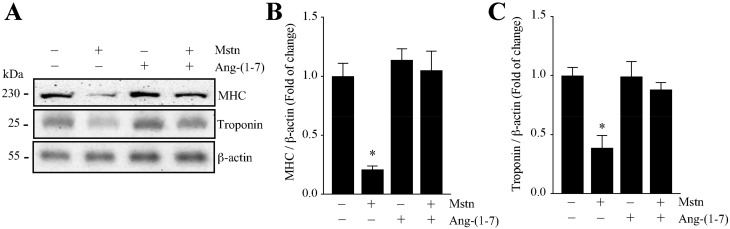
Ang-(1-7) decreased the myostatin-dependent decline in the myofibrillar protein levels in the myotubes. The C_2_C_12_ myotubes were incubated with myostatin (Mstn, 1 µg/mL) for 72 h in the presence or absence of Ang-(1-7) (10 nM; pre-incubated for 1 h). (**A**) The MHC, troponin, and β-actin protein levels were determined by Western blot analysis. The molecular weights are shown in kDa. Densitometric analysis for the MHC (**B**) and troponin (**C**) protein levels with values normalized to β-actin and expressed as a fold of change relative to the control (myotubes without treatment). All of the values correspond to the mean ± SD from three independent experiments (*n* = 3; * *p* < 0.05 vs. control without treatment).

Then, we evaluated the effect of Ang-(1-7) on the decrease of the sarcomeric protein levels induced by myostatin. Figure 2A shows the protein levels of MHC and troponin detected by Western blot analysis. The quantification of the densitometric analysis of the experiments shown in Figure 2A indicates that Ang-(1-7) prevents the myostatin-induced decrease of the MHC (Figure 2B) and troponin (Figure 2C) protein levels.

Together, these results indicate that Ang-(1-7) prevents the atrophic effect induced by myostatin in C_2_C_12_ myotubes.

### 2.2. Ang-(1-7) Decreases the Myostatin-Dependent Increment of E3 Ubiquitin Ligases and ROS in C_2_C_12_ Myotubes

**Figure 3:**
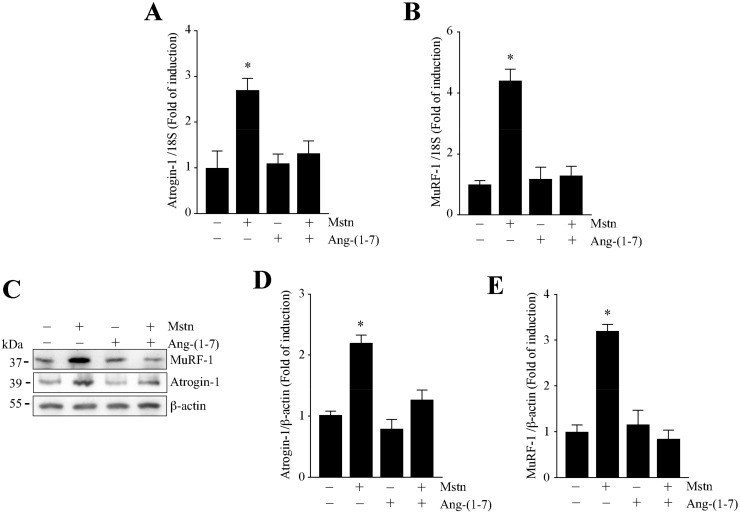
Ang-(1-7) avoided the myostatin-dependent induction of atrogin-1 and MuRF-1 in the myotubes. The C_2_C_12_ cells differentiated for five days were pretreated or not with Ang-(1-7) (10 nM) for 1 h, and further with myostatin (Mstn, 1 µg/mL) for 12 or 24 h. The mRNA levels of atrogin-1 (**A**) and MuRF-1 (**B**) were determined by RT-qPCR. (**C**) The protein levels of atrogin-1 and MuRF-1 were detected by Western Blot analysis. β-actin was used as the loading control. The molecular weights are shown in kDa. Quantitative analysis of atrogin-1 (**D**) and MuRF-1 (**E**) levels with values normalized to β-actin. All of the values correspond to the mean ± SD from three independent experiments (*n* = 3; * *p* < 0.05 vs. control without treatment).

We analyzed the effect of Ang-(1-7) on the myostatin-induced increase of atrogin-1 and MuRF-1 expression, two muscle-specific E3-ubiquitin ligases increased in muscle atrophy. First, we evaluated the gene expressions of atrogin-1 and MuRF-1. The results show that Ang-(1-7) completely abolished the increment of atrogin-1 (Figure 3A) and MuRF-1 (Figure 3B) expression induced by myostatin, reaching the basal levels.

Then, we evaluated the protein levels of atrogin-1 and MuRF-1 through Western blot analysis (Figure 3C). The quantification of these data shows that Ang-(1-7) avoids the increase of atrogin-1 (Figure 3D) and MuRF-1 (Figure 3E) expression induced by myostatin.

**Figure 4:**
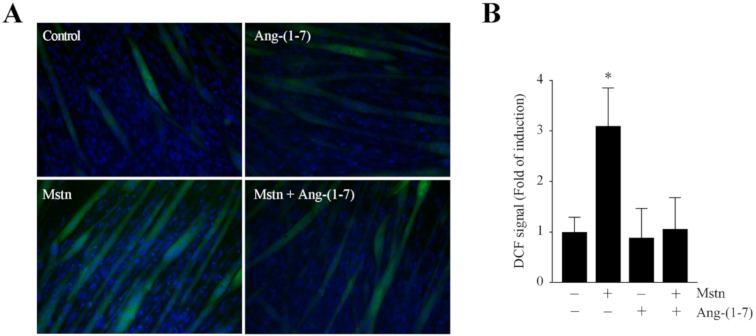
Ang-(1-7) reduced the reactive oxygen species (ROS) production induced by myostatin in the myotubes. (**A**) C_2_C_12_ myotubes were incubated with myostatin (Mstn, 1 µg/mL) for 24 h in the presence or absence of Ang-(1-7) (10 nM; pre-incubated for 1 h). The ROS levels were then determined by the detection of fluorescence using a CM-DCF-DA probe. The nuclei were labelled with Hoechst. The scale bar represents 100 µm. (**B**) Quantification of the DCF-positive myotubes. The values are expressed as a fold of induction relative to the myotubes without treatment, and correspond to the mean ± SD from three independent experiments (*n* = 3; * *p* < 0.05 vs. control without treatment).

As ROS are implicated in several forms of skeletal muscle atrophy, ROS production was assessed in the myotubes exposed to myostatin. Figure 4A shows the ROS detection through DCF-DA (dichlorodihydrofluorescein diacetate) fluorescence in myotubes incubated with myostatin in the absence or presence of Ang-(1-7). The quantification of the data indicates that Ang-(1-7) prevents the increase of myostatin-induced ROS production (3.12-fold), reaching a value similar to the basal levels (1.05-fold; Figure 4B).

These results indicate that Ang-(1-7) prevents the E3 ligase and ROS induction by myostatin in skeletal muscle cells.

### 2.3. Ang-(1-7) Decreases the NF-κB Signaling Induced by Myostatin in C_2_C_12_ Myotubes

**Figure 5:**
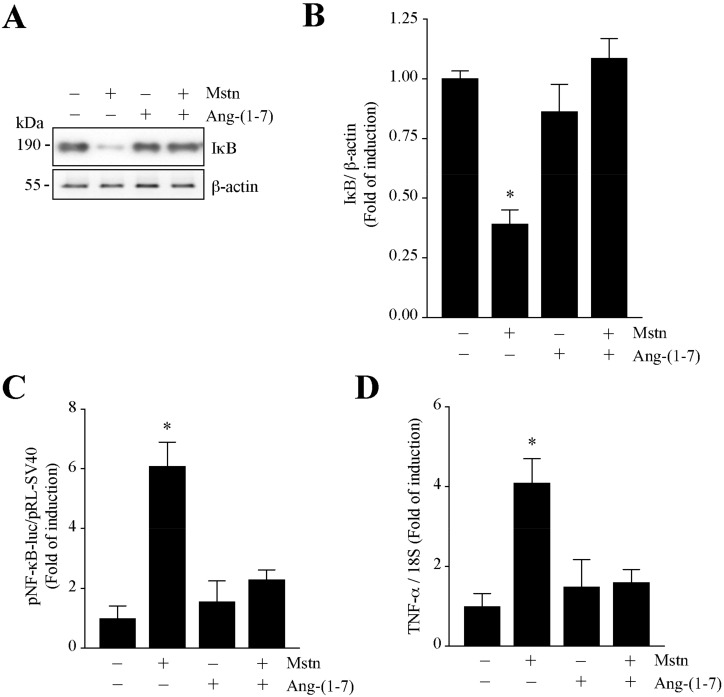
Ang-(1-7) reduced the myostatin-induced NF-κB signaling in myotubes. (**A**) C_2_C_12_ myotubes were pre-incubated with or without Ang-(1-7) (10 nM) and after 1 h with myostatin (Mstn, 1 µg/mL) for 24 h. Protein levels of IκB and β-actin were detected by Western blot analysis. Molecular weights are shown in kDa. (**B**) Quantitative analysis for IκB protein levels with values normalized to β-actin. (**C**) Luciferase activity of pNF-κB-luc. (**D**) The mRNA levels of TNF-α were determined by RT-qPCR. The values are expressed as a fold of induction relative to the control (myotubes without treatment), and correspond to the mean ± SD from three independent experiments (*n* = 3; * *p* < 0.05 vs. control without treatment).

We evaluated the modulation of the myostatin-dependent regulation of NF-κB signaling in the myotubes. First, we determined the protein levels of Iκ-B by Western blot analysis (Figure 5A). The quantification of the data indicates that myostatin decreases the protein levels of Iκ-B by 42.3% (Figure 5B). The same figure shows that Ang-(1-7) prevents the myostatin-dependent decrease of Iκ-B.

To evaluate the effects of Ang-(1-7) downstream of Iκ-B in the myostatin-induced activation of NF-κB signaling, we determined the NF-κB-dependent transcriptional activity through the plasmid reporter pNF-κB-luc (pNifty-luc) and the measurement of the gene expression of an endogenous target gene, such as TNF-α. The results show that myostatin increases the pNF-κB-luc activity (6.05-fold), which is decreased by Ang-(1-7) (2.21-fold; Figure 5C). Figure 5D shows that the myostatin-induced increment of TNF- α expression (4.15-fold) was totally abolished by Ang-(1-7) (1.41-fold).

All of these data indicate that Ang-(1-7) diminishes the myostatin-induced NF-κB signaling in C_2_C_12_ myotubes.

### 2.4. Ang-(1-7) Decreases the Myostatin-Dependent Activity through the Mas Receptor in C_2_C_12_ Myotubes

**Figure 6:**
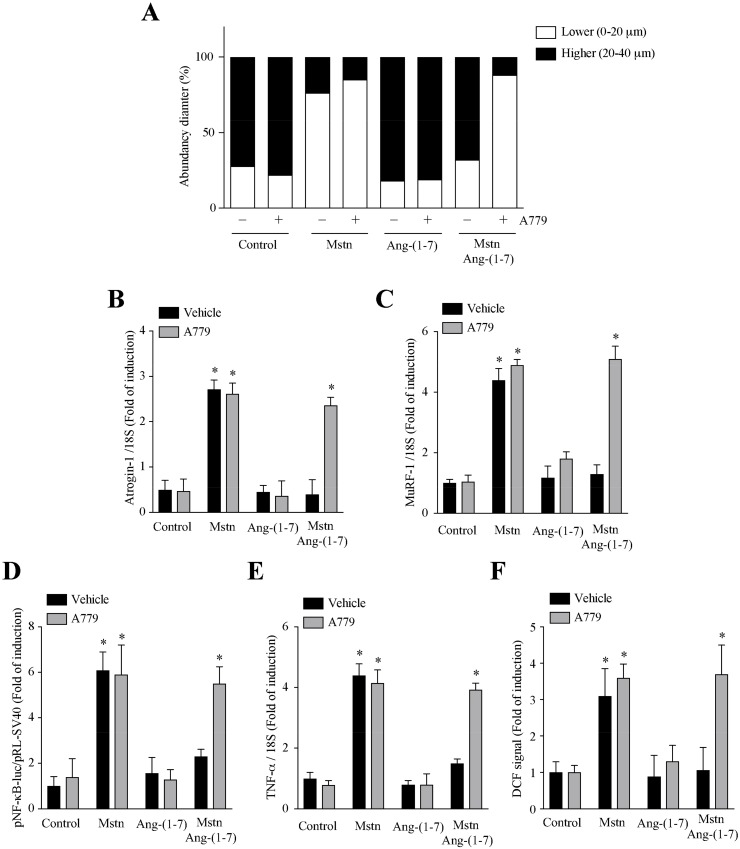
Reduction of myostatin dependent on muscle atrophy and signaling by Ang-(1-7) is mediated through Mas receptor in myotubes. C_2_C_12_ myotubes, pretreated with or without Ang-(1-7) (10 nM), A779 (10 µM) (pre-incubated for 30 min), or both, were incubated with myostatin (Mstn, 1 µg/mL) for 12 or 24 h. The parameters measured were: (**A**) distribution of the myotube diameters; mRNA levels of (**B**) atrogin-1, (**C**) MuRF-1, and (**E**) TNF-α; (**D**) Luciferase activity of pNF-κB-luc; and (**F**) ROS levels. The values are expressed as a fold of induction relative to the myotubes without treatment and correspond to the mean ± SD from three independent experiments (*n* = 3; * *p* < 0.05 vs. control without treatment).

We evaluated if the effects of Ang-(1-7) on the myostatin dependent muscle atrophy and signaling were dependent on the Mas receptor activity. For that, we used the A779 antagonist. The results show that A779 reverses the effect of Ang-(1-7) on the myotube diameter (Figure 6A) and atrogin-1 (Figure 6B), MuRF-1 (Figure 6C), and TNF-α (Figure 6E) gene expressions, as well as the pNF-κB-luc activity (Figure 6D) and ROS production (Figure 6F).

All of these data indicate that Ang-(1-7) diminishes myostatin-induced muscle atrophy and signaling through the Mas receptor in the myotubes.

### 2.5. Ang-(1-7) Decreases the Myostatin-Dependent Activity through the Akt/PKB Activity in C_2_C_12_ Myotubes

**Figure 7:**
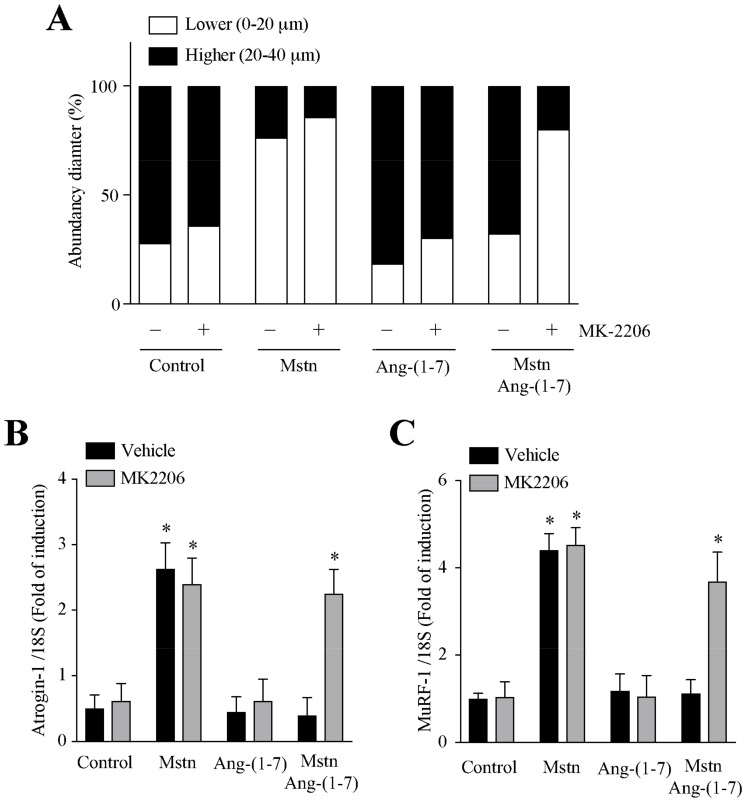

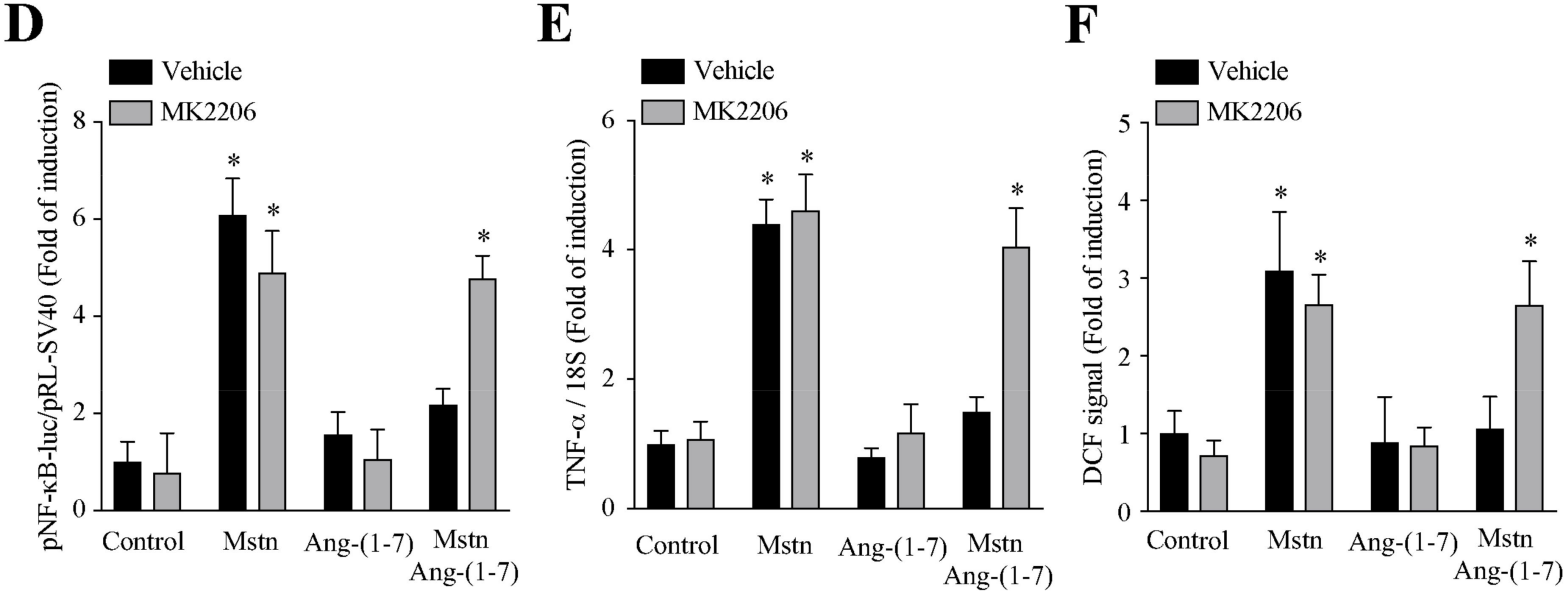
Prevention of myostatin-dependent muscle atrophy and signaling by Ang-(1-7) is mediated by the Akt/PKB activity in the myotubes. The C_2_C_12_ myotubes were incubated with myostatin (Mstn, 1 µg/mL) for 12 or 24 h in the presence or absence of Ang-(1-7) (10 nM), MK2206 (10 µM; pre-incubated for 30 min), or both. The parameters measured were as follows: (**A**) distribution of the myotube diameters; mRNA levels of (**B**) atrogin-1, (**C**) MuRF-1 and (**E**) TNF-α; (**D**) Luciferase activity of pNF-κB-luc; and (**F**) ROS levels. The values are expressed as a fold of induction relative to the myotubes without treatment, and correspond to the mean ± SD from three independent experiments (*n* = 3; * *p* < 0.05 vs. control without treatment).

As Ang-(1-7) is able to activate Akt phosphorylation in the C_2_C_12_ myotubes [26], we evaluated if the effects of Ang-(1-7) on the myostatin-dependent atrophy and signaling were dependent on the Akt activity. For this, we used the MK2206 inhibitor [26]. The results show that MK2206 prevents the effect of Ang-(1-7) on the myostatin-induced effects measured [decrease in the myotube diameter (Figure 7A) and an increase of atrogin-1 (Figure 7B), MuRF-1 (Figure 7C) and TNF-α (Figure 7E) gene expressions, as well as an increase of pNF-κB-luc activity (Figure 7D) and ROS production (Figure 7F)].

All of these data indicate that Ang-(1-7) diminishes the myostatin-induced muscle atrophy and signaling through Akt/PKB in the C_2_C_12_ myotubes.

## 3. Discussion

This study demonstrates that Ang-(1-7), via the Mas receptor, can prevent myostatin-induced atrophy. This finding is supported by an in vitro analysis of the myostatin-dependent effect on the myotube diameter, myofibrillar protein levels, MuRF-1 gene expression, and ROS production, which were abolished by Ang-(1-7). Interestingly, our results demonstrate that Ang-(1-7) also prevents the activation of NF-κB signaling induced by myostatin, and this inhibition is required for decreasing the myostatin-dependent muscle atrophy, as evaluated by the MuRF-1 gene expression.

Several data, mainly from our group and others, have demonstrated the anti-atrophic effect mediated by Ang-(1-7) on skeletal muscle both in vitro and in vivo [25,26,27,28,29,30,31,32]. This is the first report that shows that Ang-(1-7) inhibits myostatin-induced muscle atrophy. Myostatin has been described to promote the increase of protein breakdown by UPS as well as the decrease of protein synthesis [14,33,34]. Our data indicate that under myostatin stimulation, the prevention induced by Ang-(1-7) of the increased atrogin-1 and MuRF-1 expressions can probably be related to the suppression of the MHC and troponin protein level diminution, as these myofibrillar proteins are targets for the two E3 ligases, and therefore can be further degraded by UPS. Thus, under Ang-(1-7) incubation, myotubes exposed to myostatin recovered the content of the myofibrillar proteins, which could help in maintaining the myotube diameter.

Myostatin decreases the protein synthesis in skeletal muscle through a mechanism that involves the decrease of the Akt/mTOR (mammalian target of rapamycin)/p70S6K signaling pathway [35]. In this line of evidence, several reports for the crosstalk between myostatin and protein synthesis pathways have been presented [36]. Thus, the loss of function for myostatin signaling activates the protein synthesis, whereas the gain of function abolishes the protein synthesis [37,38]. Therefore, the Ang-(1-7)-mediated prevention in the diminution of the MHC and troponin levels induced by myostatin could possibly involve the increase of myofibrillar protein synthesis. In support of this idea, we have previously reported that Ang-(1-7) induces the activation of some of the mediators involved in the protein synthesis, such as IGF-1R, Akt, and p70S6K, in a model of muscle atrophy caused by disuse [25]. In this manuscript, our data indicate that the anti-atrophic effect of Ang-(1-7) myostatin-induced muscle wasting requires the Akt activity. A key protein of the protein synthesis is Akt [39,40,41]. In addition, the evidence shows that myostatin decreases Akt phosphorylation [35]. In this sense, we have previously demonstrated the Akt phosphorylation by Ang-(1-7) [26].

Myostatin has been described to increase ROS production [13]. Our findings are in agreement with this evidence. Moreover, we have demonstrated in this paper that Ang-(1-7) is able to abolish myostatin-induced ROS production. We have also previously described that TGF-β, a growth factor of the same family as myostatin, induces muscle atrophy through an ROS-dependent mechanism [42]. Moreover, Ang-(1-7) decreases the TGF- β -dependent ROS production in myotubes [28]. Other studies must be performed in order to elucidate the source of ROS production by myostatin, and the mechanism through which Ang-(1-7) decreases ROS in response to myostatin.

Ang-(1-7) also decreases the myostatin-induced activation of the NF-κB signaling pathway. Several data indicate that NF-κB signaling is a key pathway in muscle disorders, such as dystrophies and muscle atrophy [43,44,45,46]. Our data indicate that the inhibition induced by Ang-(1-7) on the myostatin-dependent NF-κB activation is necessary to decrease TNF-α gene expression. This event has high relevance, because TNF-α is an inflammatory factor widely described to produce muscle atrophy [47,48,49]. Moreover, NF-κB activation is involved in the increment of myostatin expression, which can indicate the presence of an autocrine axis in the skeletal muscle that can accentuate and perpetuate the muscle wasting.

Although Ang-(1-7) abolished the myostatin-induced NF-κB signaling activation, the mechanism has not been studied, and more analyses must be performed in order to elucidate it. In this line, the anti-NF-κB activity of Ang-(1-7) has been described in several tissues other than skeletal muscle as a counterregulatory effect to the Ang-II dependent NF-κB activation [50,51,52]. The relevance of the anti-atrophic effect of Ang-(1-7) and especially the depletion of NF-κB signaling has been discussed. Moreover, in some muscle disorders, the NF-κB inhibition has been demonstrated to improve muscle function and mass [53,54,55].

In summary, we have demonstrated that Ang-(1-7) is able to prevent the myostatin-induced muscle atrophy and signaling in C_2_C_12_ myotubes.

## 4. Materials and Methods

### 4.1. Cell Cultures

The skeletal muscle cell line C_2_C_12_ (American Type Culture Collection, NY, USA) was grown in Dulbecco’s modified eagle’s medium (DMEM) supplemented with 10% fetal serum bovine (FSB) and used for 10 passages. The cells were differentiated until day 5 by changing DMEM–FSB to DMEM supplemented with horse serum, as previously described [55,56,57,58]. The myotubes were incubated with 1 µg/ml of human recombinant myostatin (Cell Sciences Inc., MA, USA) and/or 10 nM of Ang-(1-7) (Sigma-Aldrich, USA), as indicated in each figure. The myotubes were treated with 10 µM of MK2206 (Selleckchem, TX, USA) for Akt/PKB inhibition, or 10 µM of A779 (CPC Scientific, CA, USA) antagonist for Mas receptor inhibition. In brief, the procedure was as follows: differentiated cells were pre-treated with Ang-(1-7) for 30 or 60 min, depending on the experiments, and then treated with myostatin. In the experiments that corresponded, the myotubes were incubated with A779 or MK2206 for 60 min prior to incubation with Ang-(1-7). Furthermore, the cells were subjected to evaluation 24 or 72 h after myostatin treatment, depending on the experiments.

### 4.2. Immunoblot Analysis

Protein extracts were obtained by myotubes homogenization in a radioimmunoprecipitation assay (RIPA) buffer supplemented with a cocktail of protease inhibitors and 1 mM of phenylmethylsulfonyl fluoride (PMSF). The proteins were subjected to SDS-PAGE, transferred onto polyvinylidene difluoride (PVDF) membranes (EMD Millipore, MA, USA), and probed with mouse anti-MHC (1:3000; MF-20, Developmental Studies, Hybridoma Bank, University of Iowa, IA, USA), rabbit anti-atrogin-1 (1:500), rabbit anti-MuRF-1 (1:500; ECM Biosciences, KY, USA), rabbit anti-troponin I (1:1000), rabbit anti-Iκ-B (1:5000), and rabbit anti-β-actin (1:5000; Santa Cruz Biotech., TX, USA). All of the immunoreactions were visualized by enhanced chemiluminescence (Thermo Scientific, MA, USA). Images were acquired using the Fotodyne FOTO/Analyst Luminary Workstation Systems (Fotodyne Inc., WI, USA). Densitometry analysis was determined by scanning the immunoreactive bands, and the intensity values were obtained for further analysis.

### 4.3. Immunofluorescence Microscopy

The location of the MHC was analyzed by indirect immunofluorescence (IFI) [31]. Briefly, the cells were grown and differentiated on glass coverslips and then fixed in 4% paraformaldehyde, permeabilized with 0.05% Triton X-100, and incubated for 1 h at 37 °C with 1:100 mouse anti-MHC (MF-20, Developmental Studies, Hybridoma Bank, University of Iowa, IA, USA). Afterwards, the cells were washed and incubated for 30 min at 37 °C with 1:100 affinity-purified Alexa Fluor dye-conjugated goat anti-mouse antibody (Thermo Scientific, MA, USA). For nuclear staining, the sections were incubated with 1 µg/mL Hoechst 33258 for 10 min. After rinsing, the sections were mounted with a fluorescent mounting medium (Dako Corporation, CA, USA) under glass coverslips, viewed, and photographed with the Motic BA310 epifluorescence microscope (Motic, Hong Kong).

### 4.4. Measurement of Myotube Diameter

To obtain the myotube diameters, photographs of the immunofluorescent myotubes for MHC were obtained from different zones of the coverslip. The minimal Feret diameters were measured using the ImageJ software (National Institutes of Health, MD, USA) and analyzing a total of 80 myotubes from 10 random fields from each experimental condition in a blind fashion. The myotube diameter was measured at three different points of the individual myotube, and then the values were averaged. Myotubes were defined as all multinucleated (containing three or more nuclei) cells positive for MHC stain.

### 4.5. Measurement of Intracellular ROS Levels

C_2_C_12_ myotubes cultured on glass coverslips were incubated with myostatin in the presence or absence of the different antagonists or the inhibitor for 24 h. At the end of this experiment, the cells were incubated with H2-DCF-DA for 30 min at 37 °C. After two washes with phosphate-buffered saline (PBS), the cells were fixed with 4% paraformaldehyde for 10 min and washed with PBS. The cells were incubated with 1 µg/ml Hoechst 33258 in PBS for 10 min for nuclear staining. Then, the cells were rinsed and mounted with a fluorescent mounting medium (Dako Corporation, CA, USA), viewed, and photographed with the Motic BA310 epifluorescence microscope (Motic, Hong Kong).

### 4.6. Transient Plasmid Transfection

First, 1 µg of pNifty-luc (a NF-κB-luc plasmid reporter from InvivoGen, CA, USA) and 0.02 μg of pRL-SV40 were used to co-transfect the cells using 1 μl of LipofectAMINE 3000 in Opti-MEM I. After 6 h, FBS was added to the medium, and the cells were cultured for a further 12 h. Furthermore, the cells were differentiated by 4 days, and the treatments were performed as indicated in the figures. Dual luciferase activity assays (Promega, WI, USA) were performed after 24 h in a GloMax 20/20 luminometer (Promega, WI, USA).

### 4.7. RNA Isolation, Reverse Transcription, and Quantitative Real-Time PCR

The total RNA was isolated from the cell cultures using Trizol (Thermo Scientific, MA, USA). The total RNA (1 µg) was mixed with random hexamers and SuperScript Reverse Transcriptase (Thermo Scientific, MA, USA) to reverse transcribe it to cDNA. Then, quantitative real-time PCR was performed using pre-designed primer sets for mouse atrogin-1, MuRF-1, and TNF-α as well as housekeeping gene 18 S (Taqman Assays-on-Demand, Applied Biosystems, MA, USA). All of the reactions were performed in triplicate on an Eco Real-Time PCR System (Illumina, CA, USA). The mRNA expression was quantified using the comparative ΔCt method (2-ΔΔCT). The mRNA levels are expressed relative to the mean expression in the control group. The values correspond to the mean of the ΔCt value ± standard deviation (SD) of the three independent experiments.

### 4.8. Statistics

Data were statistically analyzed using a one-way analysis of variance (ANOVA) with a post-hoc Bonferroni multiple-comparison test (GraphPad Software, Prism version 8, CA, USA). Differences were considered statistically significant at a *p*-value of <0.05.
